# Effective 1D Time-Dependent Schrödinger Equations for 3D Geometrically Correlated Systems

**DOI:** 10.3390/ma13133033

**Published:** 2020-07-07

**Authors:** Devashish Pandey, Xavier Oriols, Guillermo Albareda

**Affiliations:** 1 Departament d’Enginyeria Electrònica, Universitat Autònoma de Barcelona, Edifici Q, 08193 Bellaterra, Spain; 2 Max Planck Institute for the Structure and Dynamics of Matter and Center for Free-Electron Laser Science, Luruper Chaussee 149, 22761 Hamburg, Germany; 3 Institut de Química Teòrica i Computacional (IQTCUB), Universitat de Barcelona, Martí i Franquès 1, 08028 Barcelona, Spain

**Keywords:** nanojunction, constriction, quantum electron transport, quantum confinement, dimensionality reduction, stochastic Schrödinger equations, geometric correlations

## Abstract

The so-called Born–Huang ansatz is a fundamental tool in the context of ab-initio molecular dynamics, viz., it allows effectively separating fast and slow degrees of freedom and thus treating electrons and nuclei with different mathematical footings. Here, we consider the use of a Born–Huang-like expansion of the three-dimensional time-dependent Schrödinger equation to separate transport and confinement degrees of freedom in electron transport problems that involve geometrical constrictions. The resulting scheme consists of an eigenstate problem for the confinement degrees of freedom (in the transverse direction) whose solution constitutes the input for the propagation of a set of coupled one-dimensional equations of motion for the transport degree of freedom (in the longitudinal direction). This technique achieves quantitative accuracy using an order less computational resources than the full dimensional simulation for a typical two-dimensional geometrical constriction and upto three orders for three-dimensional constriction.

## 1. Introduction

Nanoscale constrictions (sometimes referred to as point contacts or nanojunctions) are unique objects for the generation and investigation of ballistic electron transport in solids. Studies of such systems have been inspired by the pioneering investigations of Sharvin in the mid-1960s [1]. Today, advances in fabrication techniques like direct growth of branched nanostructures [2], electron beam irradiation [3], thermal and electrical welding [4], or atomic force microscope [5] have allowed controlling the size and composition of nanojunctions for creating devices with desired functionalities. In this respect, a number of nanodevices based on nanojunctions like single electron transistors [6,7], field effect transistors [8,9], and heterostructure nanowires [10,11] have been recently reported, which promise great performance in terms of miniaturization and power consumption.

In the design of these nanostructures, simulation tools constitute a valuable alternative to the expensive and time-consuming test-and-error experimental procedure. A number of quantum electron transport simulators are available to the scientific community [12,13,14,15,16]. The amount of information that these simulators can provide, however, is mainly restricted to the stationary regime, and therefore, their predicting capabilities are still far from those of the traditional Monte Carlo solution of the semi-classical Boltzmann transport equation [17]. This limitation poses a serious problem in the near future as electron devices are foreseen to operate in the terahertz (THz) regime. At these frequencies, the discrete nature of electrons in the active region is expected to generate unavoidable fluctuations of the current that could interfere with the correct operation of such devices both for analog and digital applications [18].

A formally exact approach to electron transport beyond the quasi-stationary regime relies in the modeling of the active region of electron devices as an open quantum system [19,20]. As such, one can then borrow any state-of-the-art mathematical tool developed to study open quantum systems [21,22]. A preferred technique has been the stochastic Schrödinger equation (SSE) approach [23,24,25,26,27,28,29,30]. Instead of directly solving equations of motion for the reduced density matrix, the SSE approach exploits the state vector nature of the so-called conditional states to alleviate some computational burden [31].

As an example of the practical utility of the SSE, a Monte Carlo simulation scheme to describe quantum electron transport in open systems that is valid both for Markovian or non-Markovian regimes guaranteeing a dynamical map that preserves complete positivity has been recently proposed [32]. The resulting algorithm for quantum transport simulations reformulates the traditional “curse of dimensionality” that plagues all state-of-the-art techniques for solving the time-dependent Schrödinger equation (TDSE). Specifically, the algorithm consists of the solution of an ensemble of single-particle SSEs that are coupled, one to each other, through effective Coulombic potentials [33,34,35]. Furthermore, the simulation technique accounts for dissipation [36] and guarantees charge and current conservation through the use of self-consistent time-dependent boundary conditions [37,38,39] that partially incorporate exchange correlation [39,40]. Solving a large number of three-dimensional (3D) single-particle TDSEs, however, may still be a very time-consuming task. Therefore, the above technique would greatly benefit from the possibility of further reducing the dimensionality of the associated numerical problem.

It is the purpose of this work to derive and discuss a method that allows solving the 3D TDSE in terms of an ensemble of one-dimensional (1D) TDSEs. The technique is inspired by the so-called Born–Huang ansatz [41], which is a fundamental tool in the context of ab-initio molecular dynamics that allows separating fast and slow degrees of freedom in an effective way [42]. Here, we consider an analogous ansatz to separate transport and confinement directions. As it will be shown, the resulting technique allows to describe arbitrary geometric correlations in terms of a coupled set of 1D TDSEs. Therefore, while we have motivated the development of this method in the context of the simulation of (non-Markovian) quantum transport in open systems, the method presented here could be of great utility in many research fields where the reduction of the computational cost associated with the solution of an ensemble of SSEs may be advantageous. This includes, for example, the description of spin thermal transport [43,44], thermal relaxation dynamics [19,45], ionic motion [46,47], or Bose–Einstein condensates [48,49,50] in terms of SSEs.

The manuscript is structured as follows. In Section 2, we introduce a Born–Huang-like ansatz that allows expanding the 3D single-particle TDSE in terms of an infinite set of (transverse) eigenstates weighted by (longitudinal) complex coefficients. The equations of motion for the coefficients are found to be coupled and obey a non-unitary partial differential equation. In Section 3, we apply the method to a typical 2D constriction. Section 3.1 is devoted to finding analytical expressions for the effective potentials that appear in the equation of motion of the coefficients. A discussion on the geometrical dependence of these effective potentials is provided. In Section 3.2, we illustrate the performance of the method to describe the dynamics of an electron across a 2D nanojunction. In Section 4, we provide a thorough discussion on the advantages and potential drawbacks of the method. We conclude in Section 5.

## 2. Single-Electron Time-Dependent Schrödinger Equation in a Born–Huang-Like Basis Expansion

As we explained in the Introduction, it is our goal to reduce the computational burden associated with the solution of an ensemble of effective single-electron 3D SSE [32]. We consider our starting point to be the 3D TDSE of a single (spin-less) electron in the position basis, i.e.,

(1) $$i\frac{\partial }{\partial t}\Psi \left(x,y,z,t\right)=H\left(x,y,z\right)\Psi \left(x,y,z,t\right),$$

where we have used atomic units, *x*, *y*, and *z* represent the three spatial coordinates, and Ψ(x,y,z,t) is a well normalized wavefunction, i.e., ${\;\int \int \int \;dx\;dy\;dz\;|\Psi \left(x,y,z,t\right)|}^{2}=1\;\;\forall t$. In Equation (1), H(x,y,z) is the full Hamiltonian of the system:

(2) $$\begin{array}{ccc} H(x,y,z) & = & T_{x}+T_{y}+T_{z}+V\left(x\right)+W\left(x,y,z\right), \end{array}$$

which has been assumed to be time-independent for simplicity. The time-dependence of the scalar potentials V(x) and W(x,y,z) will be discussed in later sections. In Equation (2), $T_{x}=-\frac{1}{2}\frac{\partial^{2}}{\partial x^{2}}$ and V(x) are, respectively, the kinetic energy and the scalar potential associated with the longitudinal degree of freedom *x*, while $T_{y}=-\frac{1}{2}\frac{\partial^{2}}{\partial y^{2}}$ and $T_{z}=-\frac{1}{2}\frac{\partial^{2}}{\partial z^{2}}$ are the kinetic energies associated with the transversal degrees of freedom *y* and *z*. The scalar potential W(x,y,z) includes any other scalar potential that is not purely longitudinal, which is responsible for making the solution of Equation (1) non-separable.

It is convenient at this point to rewrite the Hamiltonian in Equation (2) in terms of longitudinal and transverse components as:

(3) $$H\left(x,y,z\right)=T_{x}+V\left(x\right)+H_{x}^{⊥}\left(y,z\right),$$

where $H_{x}^{⊥}\left(y,z\right)$ is the transverse Hamiltonian defined as:

(4) $$H_{x}^{⊥}\left(y,z\right)=T_{y}+T_{z}+W\left(x,y,z\right).$$

An eigenvalue equation associated with the transverse Hamiltonian can now be introduced as follows:

(5) $$\begin{array}{c} H_{x}^{⊥}\left(y,z\right)\phi_{x}^{k}\left(y,z\right)=E_{x}^{k}\phi_{x}^{k}\left(y,z\right), \end{array}$$

where $E_{x}^{k}$ and $\phi_{x}^{k}\left(y,z\right)$ are the corresponding eigenvalues and eigenstates respectively, and k∈Z. The eigenstates $\phi_{x}^{k}\left(y,z\right)$ form a complete orthonormal basis in which to expand the Hilbert space spanned by the variables *x*, *y*, and *z*. Therefore, the 3D wavefunction in Equation (1) can be expressed in terms of transverse eigenstates $\phi_{x}^{k}\left(y,z\right)$ as:

(6) $$\begin{array}{c} \Psi \left(x,y,z,t\right)=\sum_{k=1}^{\infty }\chi^{k}\left(x,t\right)\phi_{x}^{k}\left(y,z\right), \end{array}$$

where $\chi^{k}\left(x,t\right)=\;\int \int \;dydz\phi_{x}^{k}\left(y,z\right)\Psi \left(x,y,z,t\right)$ are complex longitudinal coefficients associated with the transverse eigenstate $\phi_{x}^{k}\left(y,z\right)$. Unless otherwise stated, all integrals are evaluated from −∞ to ∞. It is important to note that since the longitudinal variable *x* appears as a parameter in Equation (5), the transverse eigenstates obey the following normalization condition:

(7) $$\;\int \int \;dydz\phi_{x}^{l}\left(y,z\right)\phi_{x}^{k}\left(y,z\right)=\delta_{lk},\;\;\forall x.$$

In addition, since the full wavefunction Ψ(x,y,z,t) is well normalized, then the longitudinal complex coefficients $\chi^{k}\left(x,t\right)$ in Equation (6) fulfill the condition:

(8) $$\sum_{k=1}^{\infty }\int dx{\left|\chi^{k}\left(x,t\right)\right|}^{2}=1.$$

The wavefunction expansion in Equation (6) can now be introduced into Equation (1) to obtain an equation of motion for the coefficients $\chi^{k}\left(x,t\right)$ (see Appendix A):

(9) $$i\frac{\partial }{\partial t}\chi^{k}\left(x,t\right)=\left(T_{x}+E_{x}^{k}+V\left(x\right)\right)\chi^{k}\left(x,t\right)-\sum_{l=1}^{\infty }\left(S^{kl}\left(x\right)+F^{kl}\left(x\right)\frac{\partial }{\partial x}\right)\chi^{l}\left(x,t\right),$$

where $E_{x}^{k}$ are effective potential energies (that correspond to the eigenvalues in Equation (5)) and $F^{kl}\left(x\right)$ and $S^{kl}\left(x\right)$ are geometric (first and second order) coupling terms, which read:

(10a) $$F^{kl}\left(x\right)=\;\int \int \;dydz\phi_{x}^{*l}\left(y,z\right)\frac{\partial }{\partial x}\phi_{x}^{k}\left(y,z\right),$$

(10b) $$S^{kl}\left(x\right)=\frac{1}{2}\;\int \int \;dydz\phi_{x}^{*l}\left(y,z\right)\frac{\partial^{2}}{\partial x^{2}}\phi_{x}^{k}\left(y,z\right).$$

Since the transverse eigenstates $\phi_{x}^{k}\left(y,z\right)$ are the energy eigenstates of a bounded system and can always be chosen to be real, the term $F^{kk}$ is zero by construction. The other terms in Equation (10) dictate the transfer of the probability presence between different longitudinal coefficients $\chi^{k}\left(x,t\right)$ and, therefore, will be called geometric non-adiabatic couplings (GNACs). Accordingly, one can distinguish between two different dynamic regimes in Equation (9):

(i) Geometric adiabatic regime: This is the regime where $F^{kl}$ and $S^{kl}$ are both negligible. Thus, the solution of Equation (9) can be greatly simplified because it involves only one transverse eigenstate.
(ii) Geometric non-adiabatic regime: This is the regime where either or both $F^{kl}$ and $S^{kl}$ are important. Thus, the solution of Equation (9) involves the coupling between different longitudinal coefficients and hence more than one transverse eigenstate.

Interestingly, the prevalence of either the regimes (i) or (ii) can be estimated by rewriting the first order coupling terms $F^{kl}\left(x\right)$ as (see Appendix B for an explicit derivation):

(11) $$F^{kl}\left(x\right)=\frac{\int dy\int dz\phi_{x}^{*l}\left(y,z\right)\left(\frac{\partial }{\partial x}W\left(x,y,z\right)\right)\phi_{x}^{k}\left(y,z\right)}{E_{x}^{l}-E_{x}^{k}}\;\;\;\;\forall k\neq l.$$

That is, the importance of non-adiabatic transitions between transverse eigenstates depends on the interplay between the transverse potential-energy differences $E_{x}^{l}-E_{x}^{k}$ and the magnitude of the classical force field, which is proportional to $\frac{\partial }{\partial x}W\left(x,y,z\right)$. The geometric adiabatic regime (i) is reached either when the classical force field is very small or the energy differences $E_{x}^{l}-E_{x}^{k}$ are large enough. In the adiabatic regime, only the diagonal terms, $S^{kk}$, are retained, which induce a global shift of the potential-energies $E_{x}^{k}$ felt by the longitudinal coefficients $\chi^{k}\left(x,t\right)$. In this approximation, the longitudinal degree of freedom moves in the potential-energy of a single transverse state, i.e., $E_{x}^{k}$. This regime is analogous to the so-called Born–Oppenheimer approximation in the context of molecular dynamics [51], where the term $S^{kk}$ is often called the Born–Oppenheimer diagonal correction [52]. As will be shown in our numerical example, the evolution of the system can be governed either by the geometric adiabatic or nonadiabatic regime depending on the particular spatial region where the dynamics occurs.

Let us notice at this point that the time-dependence of the Hamiltonian in Equation (2) may arise either due to a purely longitudinal time-dependent scalar potential V(x,t) or through the time-dependence of the non-separable potential W(x,y,z,t). If the time-dependence is added only through V(x,t), then nothing changes in the above development. On the contrary, if a time-dependence is included in W(x,y,z,t), then the eigenstate problem in Equation (5) changes with time and so do the effective potential-energies $E_{x}^{k}$ and the first and second order GNACs $F^{kl}\left(x,t\right)$ and $S^{kl}\left(x,t\right)$. As it will be shown later, in this circumstance, Equation (5) should be solved self-consistently with Equation (9).

Before we move to a practical example implementing the above reformulation of the 3D TDSE, let us emphasize that it is the main goal of the set of coupled equations in Equation (9) to allow the evaluation of relevant observables in terms of 1D wavefunctions only. In this respect, let us take, for example, the case of the reduced probability density $\rho \left(x,t\right)=\;\int \int \;dydz\Psi^{*}\left(x,y,z,t\right)\Psi \left(x,y,z,t\right)$. Using the basis expansion in Equation (6), ρ(x,t) can be written as:

(12) $$\rho \left(x,t\right)=\sum_{k,l}^{\infty }\chi^{*l}\left(x,t\right)\chi^{k}\left(x,t\right)\;\int \int \;dydz\phi_{x}^{*l}\left(y,z\right)\phi_{x}^{k}\left(y,z\right),$$

and using the condition in Equation (7), the above expression reduces to:

(13) $$\rho \left(x,t\right)=\sum_{k=1}^{\infty }{\left|\chi^{k}\left(x,t\right)\right|}^{2}.$$

Therefore, according to Equation (13), the reduced (longitudinal) density is simply the sum of the absolute squared value of the longitudinal coefficients $\chi^{k}\left(x,t\right)$, which is in accordance with Equation (8), i.e., $\int dx\rho \left(x,t\right)=\sum_{k=1}^{\infty }\int dx{\left|\chi^{k}\left(x,t\right)\right|}^{2}=1$. Similarly, other relevant observables, such as the energy can easily be derived using the expansion in Equation (6) (see Appendix C).

## 3. Application of the Method to a Typical Constriction

The above formulation can be cast in the form of a numerical scheme to solve the 3D TDSE, which we will call, hereafter, geometrically correlated 1D TDSE (GC-TDSE). The scheme can be divided into two different parts corresponding to their distinct mathematical nature. The first part involves the solution of the eigenvalue problem in Equation (5), which allows evaluating the transverse eigenstates $\phi_{x}^{k}\left(y,z\right)$ and eigenvalues $E_{x}^{k}$, as well as geometric non-adiabatic couplings $F^{kl}\left(x\right)$ and $S^{kl}\left(x\right)$ in Equation (10). These quantities are required in the second part of the algorithm to solve the equation of motion of the longitudinal coefficients $\chi^{k}\left(x,t\right)$ in Equation (9), which ultimately allow us to evaluate the observables of interest.

In what follows, we discuss these two aspects of the algorithm for a typical 2D geometric constriction whose geometry does not change in time. We consider one degree of freedom in the transport direction and one degree of freedom in the transverse (or confinement) direction. The generalization to a 3D system, i.e., with two transverse degrees of freedom, is straightforward and does not add any physical insight with respect to the 2D case. As will be shown, the transverse eigenstates and eigenvalues, as well as the geometric non-adiabatic couplings are, for a time-independent constriction, functions that are computed only once. That is, the effective potential-energies $E_{x}^{k}$ and the non-adiabatic couplings $F^{kl}$ and $S^{kl}$ are computed only at the very beginning of the GC-TDSE propagation scheme. For more general time-dependent constrictions, possibly with no analytical form of W(x,y,z,t), the only change in the algorithm is that Equation (5) has to be solved, self-consistently, together with Equation (9) at each time step.

### 3.1. Evaluation of Transverse Eigenstates (and Values) and Geometric Non-Adiabatic Couplings

Let us consider the case of a 2D nanojunction represented by the scalar potential:

(14) $$V\left(x,y\right)=\left\{\begin{array}{cc} 0, & \mathrm{if}\;\;L_{2}\left(x\right)<y<L_{1}\left(x\right)\\ \infty , & \mathrm{otherwise} \end{array}\right.$$

where $L_{1}\left(x\right)$ and $L_{2}\left(x\right)$ define the shape of the constriction. The corresponding 2D Hamiltonian reads

(15) $$H\left(x,y\right)=T_{x}+V\left(x\right)+H_{x}^{⊥}\left(y\right),$$

where $H_{x}^{⊥}\left(y\right)=T_{y}+W\left(x,y\right)$. The wavefunction for a 2D constriction in terms of the Born–Huang expansion can be written as $\Psi \left(x,y,t\right)=\sum_{k}\chi^{k}\left(x,t\right)\phi_{x}^{k}\left(y\right)$, and the transverse states $\phi_{x}^{k}\left(y\right)$ are solutions of a free particle in a 1D box whose width depends on the longitudinal variable *x*, i.e.,

(16) $$\phi_{x}^{k}\left(y\right)=\left\{\begin{array}{cc} \sqrt{\frac{2}{L(x)}}\sin\left(\frac{k\pi (y-L_{2}\left(x\right))}{L(x)}\right), & \mathrm{if}\;\;L_{2}\left(x\right)<y<L_{1}\left(x\right)\\ 0, & \mathrm{otherwise}. \end{array}\right.$$

The associated eigenvalues are given by:

(17) $$E_{x}^{k}=\frac{k^{2}\pi^{2}}{2L^{2}\left(x\right)},$$

where we have defined $L\left(x\right)=L_{1}\left(x\right)-L_{2}\left(x\right)$. These energies, parametrically dependent on the longitudinal variable *x*, define the effective potential-energies on which the coefficients $\chi^{k}\left(x,t\right)$ evolve.

**Figure 1:**
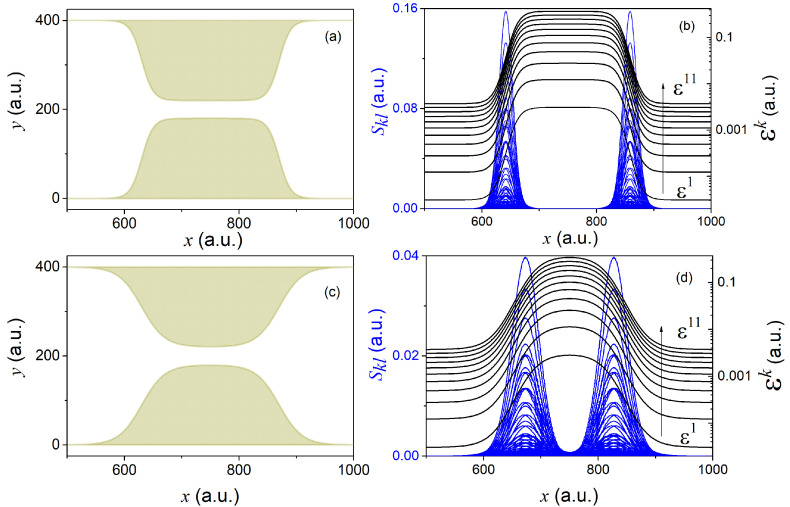
Two different nanojunctions, viz., (**a**,**c**), defined by Equation (A18a,b) in Appendix D and using A=180, B=220, $a_{1}=630$, $a_{2}=870$, with γ=10 in (**a**) and γ=20 in (**c**). Panels (**b**,**d**) show the associated second order (non-adiabatic) couplings $S^{kl}$ (solid blue lines) and the associated potential-energies $E_{x}^{k}$ (solid black lines) for the geometries in (**a**,**c**), respectively. Note that, due to the symmetry of the states defined in Equation (16), the coupling between odd and even states is zero, i.e., $F^{kl}\left(x\right)=S^{kl}\left(x\right)=0,\;\forall k+l=\mathrm{odd}$.

To evaluate the first and second order coupling terms $F^{kl}\left(x\right)$ and $S^{kl}\left(x\right)$, we need to rely on a particular form of the constriction. Depending on the specific form of $L_{1}\left(x\right)$ and $L_{2}\left(x\right)$, different constrictions can be conceived (see for example panels (a) and (c) of Figure 1). Given the states in Equation (16) and a particular shape of the constriction (defined in Equation (A18a,b) of Appendix D), it is then easy to evaluate the non-adiabatic coupling terms $F^{kl}$ and $S^{kl}$ (see panels (b) and (d) of Figure 1).

The two different constrictions in Figure 1 serve well to gain some insight into the general form and dependence of the effective potential-energies $E_{x}^{k}$, as well as of the GNACs in Equation (10). Geometries changing more abruptly lead to sharper effective potential-energies $E_{x}^{k}$ and more peaked non-adiabatic coupling terms $F^{kl}\left(x\right)$ and $S^{kl}\left(x\right)$. Sharper constrictions are thus expected to cause larger non-adiabatic transitions and hence to involve a larger number of transverse eigenstates requiring a larger number of longitudinal coefficients in order to reconstruct the reduced (longitudinal) density in Equation (12). On the contrary, smoother constrictions should yield softer non-adiabatic transitions and hence involve a smaller number of transverse eigenstates.

### 3.2. Time-Dependent Propagation of the Longitudinal Coefficients

Given the effective potential-energies $E_{x}^{k}$ and the non-adiabatic couplings $F^{kl}\left(x\right)$ and $S^{kl}\left(x\right)$, one can then easily find a solution for the longitudinal coefficients in Equation (9). Here, we consider the dynamics of an electron that impinges upon a constriction defined by Equations (14) and (A18a,b) in Appendix D using the particular set of parameters, $\left\{A,B,a_{1},a_{2},\gamma \right\}=\left\{180,220,630,870,20\right\}$, which corresponds to panel (c) of Figure 1. Due to the symmetry of the states defined in Equation (16), transitions between odd and even states are forbidden, i.e.,

(18) $$F^{kl}\left(x\right)=S^{kl}\left(x\right)=0\;\;\;\;\forall k+l=\mathrm{odd}.$$

**Figure 2:**
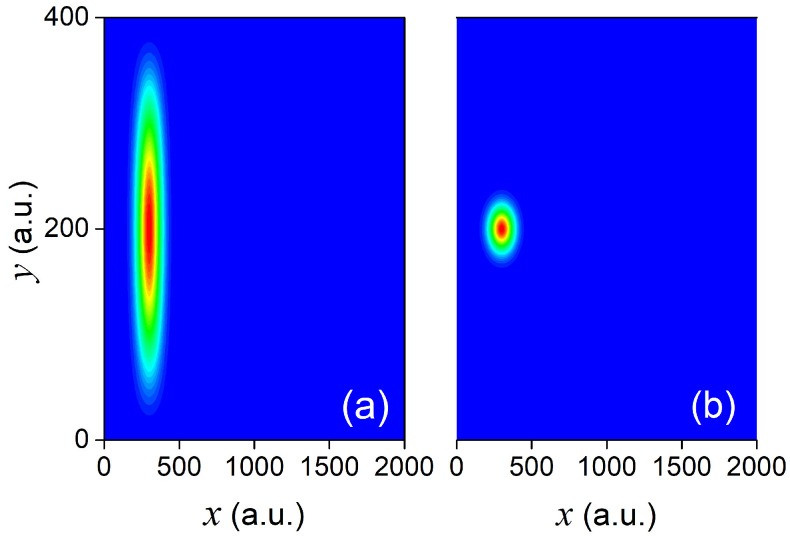
Panels (**a**,**b**) represent the probability density ${\left|\Psi \left(x,y,0\right)\right|}^{2}$ associated with the wavefunctions in Equations (19) and (20) respectively. Red regions in the plots correspond to higher probability densities, while blue regions correspond to lower probabilities.

We will consider two different initial states. On the one hand, the initial wavefunction Ψ(x,y,0) will be described by:

(19) $$\Psi \left(x,y,0\right)=\phi_{x}^{1}\left(y\right)\psi \left(x\right),$$

where $\phi_{x}^{1}\left(y\right)$ is the transverse ground state defined in Equation (16), and $\psi \left(x\right)=N\exp\left(ik_{0}\left(x-x_{0}\right)\right)\exp\left(\frac{-{\left(x-x_{0}\right)}^{2}}{2\sigma_{x}^{2}}\right)$ is a minimum uncertainty (Gaussian) wave packet with initial momentum and dispersion $k_{0}=0.086$ a.u. and $\sigma_{x}=80$ a.u., respectively, and centered at $x_{0}=300$ a.u. (while N is a normalization constant). On the other hand, we will consider the initial state to be defined by:

(20) Ψ(x,y,0)=ξ(y)ψ(x),

where now both ξ(y) and ψ(x) (defined above) are Gaussian wave packets. In particular, $\xi \left(y\right)=M\exp\left(\frac{-{\left(y-y_{0}\right)}^{2}}{2\sigma^{2}}\right)$ with $y_{0}=200$ a.u., $\sigma_{y}=20$ a.u. (and M a normalization constant). The probability densities ${\left|\Psi \left(x,y,0\right)\right|}^{2}$ associated with the above two initial states can be seen, respectively, in panels (a) and (b) of Figure 2. Given the initial states in Equations (19) and (20), we can then evaluate the corresponding longitudinal coefficients as follows:

(21) $$\chi^{k}\left(x,0\right)=\int dy\phi_{x}^{k}\left(y\right)\Psi \left(x,y,0\right).$$

While the initial state in Equation (19) corresponds to $\chi^{k}\left(x,0\right)=\delta_{k1}\psi \left(x\right)$, the state in Equation (20) involves a number of transverse eigenstates. Note that this second initial condition may be more realistic in practical situations, as large enough reservoirs may imply a quasi-continuum of transverse states according to Equation (17).

**Figure 3:**
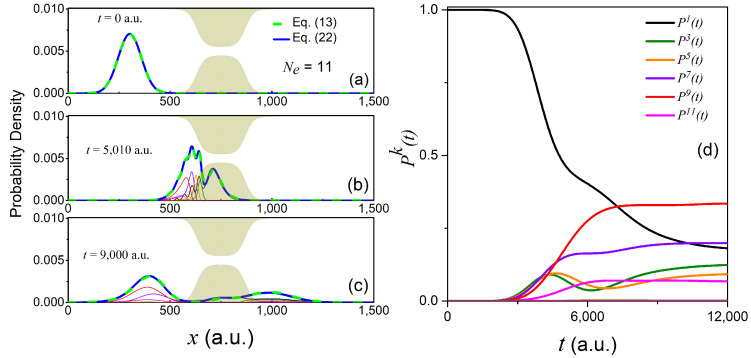
Time-evolution of the initial wavefunction in Equation (19). The reduced density in Equation (13) (dashed green line), as well as the reduced density in Equation (22) for $N_{e}=11$ (solid dark blue) are shown at times t=300 a.u., t=5010 a.u., and t=9000 a.u. in panels (**a**), (**b**), and (**c**), respectively. The rest of the lines correspond to the absolute squared value of the longitudinal coefficients $\chi^{k}\left(x,t\right)$. The evolution of the adiabatic populations in Equation (23) can be found in panel (**d**), using the same color code as in panels (**a**–**c**).

**Figure 4:**
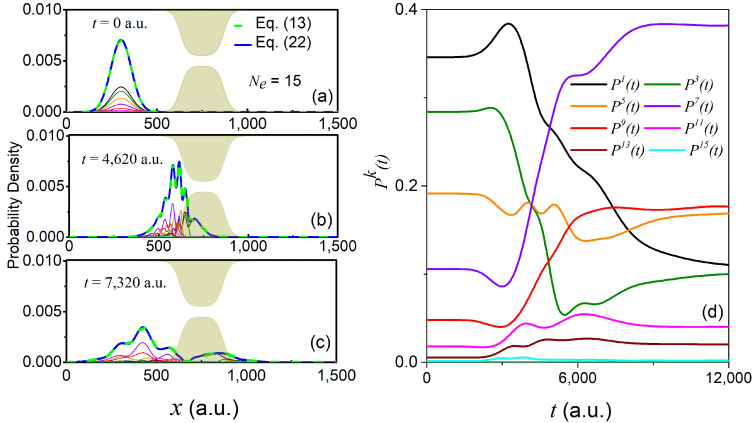
Time-evolution of the initial wavefunction in Equation (20). The reduced density in Equation (13) (dashed green line), as well as the reduced density in Equation (22) for $N_{e}=15$ (solid dark blue line) are shown at times t=0 a.u., t=4620 a.u., and t=7320 a.u. in panels (**a**), (**b**), and (**c**), respectively. The rest of the lines correspond to the absolute squared value of the longitudinal coefficients $\chi^{k}\left(x,t\right)$. The evolution of the adiabatic populations in Equation (23) can be found in panel (**d**), using the same color code as in panels (**a**–**c**).

Starting either from Equation (19) or (20), we then propagate the resulting longitudinal coefficients at the initial time according to Equation (9). Specifically, we used a fourth order Runge–Kutta method with a time-step size of Δt=0.1 a.u. and a spatial grid of 1500 points with a grid spacing Δx=1 a.u. In the left panels of Figure 3 and Figure 4, we show the time-dependent reduced density of Equation (13) evaluated from the full 2D wavefunction (dashed green line), as well as the reduced density ρ(x,t) evaluated using the GC-TDSE scheme for a finite number of transversal states $N_{e}$, i.e.,

(22) $$\rho \left(x,t\right)=\sum_{k=1}^{N_{e}}{\left|\chi^{k}\left(x,t\right)\right|}^{2}.$$

In addition, we also show the absolute squared value of the longitudinal coefficients, i.e., $|\chi^{k}{\left(x,t\right)|}^{2}$, evaluated using the GC-TDSE. Alternatively, in the right panels of Figure 3 and Figure 4, we plot the population of each transverse state,

(23) $$P^{k}\left(t\right)=\int dx{\left|\chi^{k}\left(x,t\right)\right|}^{2},$$

as a function of time using the GC-TDSE.

The initial state in Equation (19) yields $P^{k}\left(0\right)=\delta_{k1}$. This can be seen in the right-hand panel of Figure 3. This value stays constant until the wave packet hits the constriction at around t=2500 a.u. At this moment, non-adiabatic transitions between different transverse states start to occur that lead to complicated interference patterns at later times (see, e.g., the reduced density ρ(x,t) at t=5010 a.u.). The number of significantly populated transverse states increases up to six (while up to eleven states are required to reproduce the exact reduced density up to a 0.1% error). Among these states, only odd transverse states are accessible due to the symmetry of the initial state (as we noted in Equation (18)). Since the mean energy of the initial state in Equation (19) ($\langle \hat{E}\rangle =0.0037$ a.u.) is higher than the barrier height of the first effective potential-energy in Figure 1d ($\max(E_{x}^{1})=0.0028$ a.u.), one could naively expect a complete transmission of the wave packet $\chi^{1}\left(x,t\right)$. However, due to the effect of the non-adiabatic coupling terms $F^{kl}\left(x\right)$ and $S^{kl}\left(x\right)$, $\chi^{1}\left(x,t\right)$ loses a major part of its population in favor of higher energy transverse components that are reflected by much higher effective potential-energy barriers.

Starting with the second initial state in Equation (20), there are up to seven transverse states populated at the initial time, all of which are again odd states due to the symmetry of the initial conditions (see Figure 4d). Once the wave packet hits the constriction at t=3000 a.u, States 3, 7, and 9 become more dominant than the previously dominant States 1, 3, and 5. Overall, up to 15 states become important to reproduce the exact reduced longitudinal density within a 0.1% error. Given the above two examples, it seems clear that the specific form of the impinging wavefunction does play a significant role in the scaling of the number of relevant transverse states $N_{e}$ required to evaluate Equation (9) numerically.

**Figure 5:**
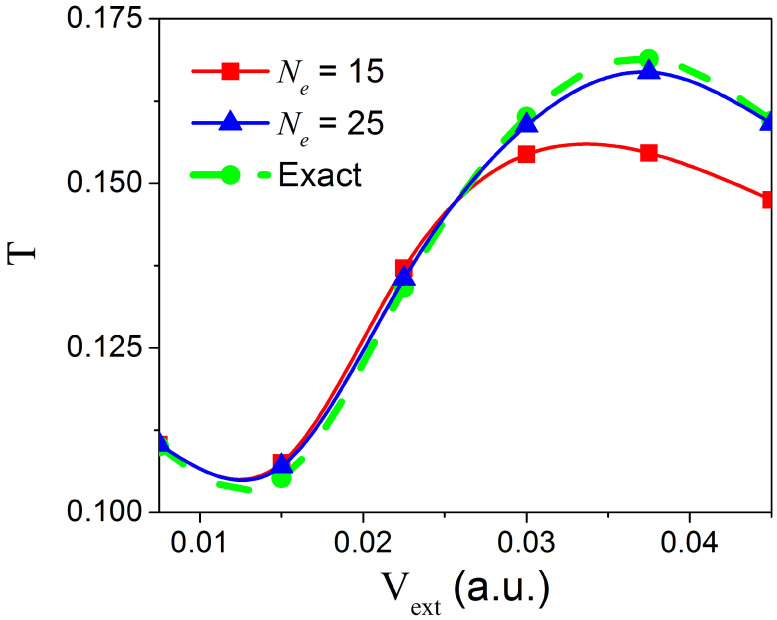
Figure depicting the transmission coefficient, *T*, under different bias voltages applied along the longitudinal direction. This plot provides a comparison for *T* between the exact 2D simulation (shown in dashed green line) and the 1D simulation (shown in solid red line for $N_{e}=15$ states and in the solid blue line for $N_{e}=25$ states). For a voltage range of $0.0075\;a.u.\leq V_{ext}\leq 0.045\;a.u.$, $N_{e}=25$ states are enough to capture the exact 2D case. For $\max[V_{ext}]=0.027$ a.u., $N_{e}=15$ states sufficiently capture the exact 2D case.

Let us finally consider the effect that an external bias along the longitudinal direction might have on the number $N_{e}$ of transverse eigenstates required to reproduce the solution of the full 2D TDSE. For that, starting with the state in Equation (20), we consider the transmission coefficient $T=\int_{-\infty }^{\infty }dy\int_{x_{m}}^{\infty }dx{\left|\Psi \left(x,y,t_{f}\right)\right|}^{2}$ for different values of the external potential $V_{ext}=V\left(x\right)$ in Equation (15). Written in terms of the Born–Huang expansion in Equation (6), the transmission coefficient *T* reads:

(24) $$T=\sum_{k=1}^{N_{e}}\int_{x_{m}}^{\infty }dx{\left|\chi^{k}\left(x,t_{f}\right)\right|}^{2},$$

where $x_{m}$ is the center of the nanojunction in the longitudinal direction (i.e., with numerical value 750 a.u.) and $t_{f}$ is the time at which no probability density (i.e., less than 0.1%) remains inside the constriction. In Figure 5, we show results for applied bias $0.0075\;a.u.\leq V_{ext}\leq 0.045\;a.u.$ and two different number of states $N_{e}=15$ and $N_{e}=25$. As expected, a higher applied bias leads to a more vigorous collision of the wave packet against the constriction due to a higher longitudinal momentum/energy, which allows higher energy transverse states to be populated.

## 4. General Discussion

The GC-TDSE algorithm discussed in the previous sections has a clear computational advantage over the solution of the full 2D TDSE. This is particularly so when the quantities $E_{x}^{k}$, $F^{kl}\left(x\right)$, and $S^{kl}\left(x\right)$, involved in the equation of motion of the longitudinal coefficients $\chi^{k}\left(x,t\right)$, are time-independent functions. For a time-independent transverse Hamiltonian, the quantities $E_{x}^{k}$, $F^{kl}\left(x\right)$, and $S^{kl}\left(x\right)$ are computed only once before the propagation of the longitudinal coefficients, and thus, the computational cost of the GC-TDSE resides, mainly, on the propagation of the 1D longitudinal coefficients.

Let us provide some numbers to estimate the numerical efficiency of the GC-TDSE algorithm. Consider the numerical solution of the full 2D TDSE in a grid. For a number of grid points $\left\{n_{x},n_{y}\right\}=\left\{1500,400\right\}$, the resulting Hamiltonian has a dimension ${\left(n_{x}\times n_{y}\right)}^{2}$. Alternatively, the size of the Hamiltonian involved in the propagation of the longitudinal coefficients of the GC-TDSE algorithm is ${\left(n_{x}\times N_{e}\right)}^{2}$, where $N_{e}$ is the number of transverse eigenstates. One can then estimate the numerical efficiency of one method over the other by simply evaluating the ratio ${\left(n_{x}\times n_{y}\right)}^{2}/{\left(n_{x}\times N_{e}\right)}^{2}$. Thus, for time-independent transverse potentials W(x,y,z), the computational reduction associated with the GC-TDSE is $n_{y}^{2}/N_{e}^{2}$. Note that the benefits of the GC-TDSE would be even more noticeable when applied to a 3D problem, for which the above ratio would become $\left(n_{y}^{2}\times n_{z}^{2}\right)/N_{e}^{2}$.

As we have seen in the above section, the number of required transverse states $N_{e}$ is a function of the abruptness/smoothness of the constriction, but also of the energy of the impinging wave packet. Therefore, the computational advantage of the GC-TDSE method over the full dimensional TDSE is clearly system-dependent. Slow wave packets impinging upon smooth constrictions would maximize the benefits of the GC-TDSE. Contrarily, very energetic electrons colliding against abrupt constrictions would certainly minimize its benefits. In this respect, we must note that the GNACs have a clear dependence on the profile of the constriction. In particular, the second order coupling terms $S^{kl}\left(x\right)$ will be sharply peaked for very abrupt constrictions (see the important differences in the size and sharpness of the $S^{kl}\left(x\right)$ in Figure 1 for two different constrictions). Therefore, due to the non-unitary character of the equations of motion of the longitudinal coefficients, very abrupt constrictions may demand very fine grids in practice.

Finally, let us mention that whenever the transverse Hamiltonian in Equation (4) is time-dependent, the advantage of the GC-TDSE method compared to the solution of the full dimensional TDSE is not so obvious. As we have already noticed, for a time-dependent transverse potential W(x,y,z,t), the eigenvalue problem in Equation (5) must be solved self-consistently with Equation (9), i.e., at each time step. Then, a comparison of the GC-TDSE and the full dimensional TDSE in terms of numerical efficiency will depend on the specific performance of the eigensolver utilized to evaluate the transverse eigenvalues, $E_{x}^{k}$, and eigenstates $\phi_{x}^{k}\left(y,z\right)$.

## 5. Conclusions

In this work, we proposed a new method, named GC-TDSE, that allows including arbitrary 3D geometric correlations between traversal and longitudinal degrees of freedom into a coupled set of 1D TDSE. Our motivation for the development of this method was, initially, the reduction of the dimensionality of the 3D Schrödinger-like equations that result from an SSE (Monte Carlo) approach to quantum electron transport in open systems (valid for Markovian and non-Markovian regimes) that we recently proposed [32]. Nevertheless, the method presented here is general and allows reducing the dimensionality of quantum systems with geometrical correlations among different degrees of freedom, which could be of utility also in different research fields such as for example spin thermal transport [43,44], thermal relaxation dynamics [19,45], ionic motion [46,47], or Bose–Einstein condensates [48,49,50].

For smooth time-independent constriction profiles under low applied bias, our GC-TDSE method implies up to three orders of magnitude less computational resources than solving the full 3D TDSE directly. For very high applied bias or time-dependent constriction profiles, the GC-TDSE may still be significantly less expensive than the solution of the full 3D TDSE, but would require introducing approximations to the solution of the potential-energies $E_{x}^{k}$ and the GNACs ($F^{kl}\left(x,t\right)$ and $S^{kl}\left(x,t\right)$). We thus expect the GC-TDSE presented here to trigger future investigation for making it robust against stronger geometrical correlations among different spatial directions. 

## Appendix A. Derivation of Equation (9)

In order to derive Equation (9), we start by introducing Equation (6) into Equation (1) to get:

(A1) $$i\frac{\partial }{\partial t}\sum_{k}\chi^{k}\left(x,t\right)\phi_{x}^{k}\left(y,z\right)=T_{x}\sum_{k}\chi^{k}\left(x\right)\phi_{x}^{k}\left(y,z\right)+H\left(y,z\right)\sum_{k}\chi^{k}\left(x\right)\phi_{x}^{k}\left(y,z\right)+V\left(x\right)\sum_{k}\chi^{k}\left(x\right)\phi_{x}^{k}\left(y,z\right).$$

Making use of Equation (5), the above equation can be written as:

(A2) $$i\frac{\partial }{\partial t}\sum_{k}\chi^{k}\left(x,t\right)\phi_{x}^{k}\left(y,z\right)=T_{x}\sum_{k}\chi^{k}\left(x\right)\phi_{x}^{k}\left(y,z\right)+E_{x}^{k}\sum_{k}\chi^{k}\left(x\right)\phi_{x}^{k}\left(y,z\right)+V\left(x\right)\sum_{k}\chi^{k}\left(x\right)\phi_{x}^{k}\left(y,z\right).$$

By simply realizing that $T_{x}=-\frac{1}{2}\frac{\partial^{2}}{\partial x^{2}}$ is the kinetic energy operator in the position basis, we expand the term $T_{x}\sum_{k}\chi^{k}\left(x\right)\phi_{x}^{k}\left(y,z\right)$ as:

(A3) $$\begin{array}{ccc} -\frac{1}{2}\frac{\partial^{2}}{\partial x^{2}}\sum_{k}\left(\chi^{k}\left(x,t\right)\phi_{x}^{k}\left(y,z\right)\right) & = & -\frac{1}{2}\frac{\partial }{\partial x}\sum_{k}\left(\frac{\partial \chi^{k}\left(x,t\right)}{\partial x}\phi_{x}^{k}\left(y,z\right)+\chi^{k}\left(x,t\right)\frac{\partial }{\partial x}\phi_{x}^{k}\left(y,z\right)\right)\\ & = & -\frac{1}{2}\sum_{k}\left(\frac{\partial^{2}}{\partial x^{2}}\left(\chi^{k}\left(x,t\right)\right)\phi_{x}^{k}\left(y,z\right)+2\frac{\partial }{\partial x}\chi^{k}\left(x,t\right)\frac{\partial }{\partial x}\phi_{x}^{k}\left(y,z\right)+\chi^{k}\left(x,t\right)\frac{\partial^{2}}{\partial x^{2}}\phi_{x}^{k}\left(y,z\right)\right), \end{array}$$

which can be written in a more compact way as:

(A4) $$T_{x}\sum_{k}\chi^{k}\left(x,t\right)\phi_{x}^{k}\left(y,z\right)=\sum_{k}\left[\left(T_{x}\chi^{k}\left(x,t\right)\right)\phi_{x}^{k}\left(y,z\right)-\frac{1}{2}\chi^{k}\left(x,t\right)\frac{\partial^{2}}{\partial x^{2}}\phi_{x}^{k}\left(y,z\right)-\frac{\partial }{\partial x}\chi^{k}\left(x,t\right)\frac{\partial }{\partial x}\phi_{x}^{k}\left(y,z\right)\right].$$

Now, introducing Equation (A4) back into Equation (A2), we get:

(A5) $$\begin{array}{ccc} i\frac{\partial }{\partial t}\sum_{k}\chi^{k}\left(x,t\right)\phi_{x}^{k}\left(y,z\right) & = & \sum_{k}\left[T_{x}+E_{x}^{k}\left(y,z\right)+V\left(x\right)\right]\chi^{k}\left(x,t\right))\phi_{x}^{k}\left(y,z\right)\\ & - & \frac{1}{2}\sum_{k}\left[\chi^{k}\left(x,t\right)\frac{\partial^{2}}{\partial x^{2}}\phi_{x}^{k}\left(y,z\right)+2\frac{\partial }{\partial x}\chi^{k}\left(x,t\right)\frac{\partial }{\partial x}\phi_{x}^{k}\left(y,z\right)\right] \end{array}$$

Multiplying both sides of Equation (A5) by $\int dy\int dz\phi_{x}^{*l}\left(y,z\right)$ and using the orthogonality condition, $\int dy\int dz\phi_{x}^{*l}\left(y,z\right)\phi_{x}^{k}\left(y,z\right)=\delta_{k,l}$, we finally obtain:

(A6) $$\begin{array}{c} i\frac{\partial }{\partial t}\chi^{k}\left(x,t\right)=\left(T_{x}+E_{x}^{k}+V\left(x\right)\right)\chi^{k}\left(x,t\right)-\sum_{l=1}^{\infty }\left(S^{kl}\left(x\right)+F^{kl}\left(x\right)\frac{\partial }{\partial x}\right)\chi^{l}\left(x,t\right), \end{array}$$

where we have defined $S^{kl}=\frac{1}{2}\int dy\int dz\phi_{x}^{*l}\left(y,z\right)\frac{\partial^{2}}{\partial x^{2}}\phi_{x}^{k}\left(y,z\right)$ and $F^{kl}=\int dy\int dz\phi_{x}^{*l}\left(y,z\right)\frac{\partial }{\partial x}\phi_{x}^{k}\left(y,z\right)$ as the first and second order coupling terms, respectively.

## Appendix B. Derivation of Equation (11)

Let us define the wavefunctions $|\phi_{l}\rangle$ and $|\phi_{k}\rangle$. Now, evaluate the derivative of their inner product as follows,

(A7) $${\langle \phi_{l}|\phi_{k}\rangle }^{\prime }=\langle \phi_{l}^{\prime }|\phi_{k}\rangle +\langle \phi_{l}|\phi_{k}^{\prime }\rangle =\delta_{l.k}=0,\;\;k\neq l$$

This implies,

(A8) $$\langle \phi_{l}^{\prime }|\phi_{k}\rangle =-\langle \phi_{l}|\phi_{k}^{\prime }\rangle$$

Evaluating the expression given below,

(A9) $$\langle \phi_{l}|H^{⊥}|\phi_{k}\rangle^{\prime }=\langle \phi_{l}^{\prime }|H^{⊥}|\phi_{k}\rangle +\langle \phi_{l}|{\left(H^{⊥}\right)}^{\prime }|\phi_{k}\rangle +\langle \phi_{l}|H^{⊥}|\phi_{k}^{\prime }\rangle$$

where we have used the chain rule of differentiation. Using the eigenstate-eigenvalue relation from Equation (5), we get,

(A10) $$\langle \phi_{l}|H_{x}^{⊥}|\phi_{k}\rangle^{\prime }=E^{k}\langle \phi_{l}^{\prime }|\phi_{k}\rangle +\langle \phi_{l}|{\left(H^{⊥}\right)}^{\prime }|\phi_{k}\rangle +E^{l}\langle \phi_{l}|\phi_{k}^{\prime }\rangle$$

Using the relation in Equation (A8) in Equation (A10), we get,

(A11) $$\langle \phi_{l}|H^{⊥}|\phi_{k}\rangle^{\prime }=\left(E^{l}-E^{k}\right)\langle \phi_{l}|\phi_{k}^{\prime }\rangle +\langle \phi_{l}|{\left(H^{⊥}\right)}^{\prime }|\phi_{k}\rangle =0$$

In the above equation, we have made use of the fact that $\langle \phi_{l}|H^{⊥}{|\phi_{k}\rangle }^{\prime }={\left(E^{k}\langle \phi_{l}|\phi_{k}\rangle \right)}^{\prime }=0$, when k≠l Therefore,

(A12) $$\begin{array}{ccc} F^{kl}={\langle \phi_{l}|(\phi_{k}\rangle )}^{\prime } & = & \frac{\langle \phi_{l}|{\left(H^{⊥}\right)}^{\prime }|\phi_{k}\rangle }{E^{k}-E^{l}} \end{array}$$

which can be written in the position representation and using the same nomenclature as used in the main text as follows,

(A13) $$F^{kl}\left(x\right)=\int dy\int dz\phi_{x}^{*l}\left(y,z\right)\frac{\partial }{\partial x}\phi_{x}^{k}\left(y,z\right)=\frac{\int dy\int dz\phi_{x}^{*l}\left(y,z\right)\left(\frac{\partial }{\partial x}H_{x}^{⊥}\left(y,z\right)\right)\phi_{x}^{k}\left(y,z\right)}{E_{x}^{l}-E_{x}^{k}}$$

Using Equation (4), it is easy to see that only the potential function W(x,y,z) will survive after the partial derivative with respect to the variable *x*. Therefore, we can equivalently write Equation (A13) as,

(A14) $$F^{kl}\left(x\right)=\frac{\int dy\int dz\phi_{x}^{*l}\left(y,z\right)\left(\frac{\partial }{\partial x}W\left(x,y,z\right)\right)\phi_{k}\left(y,z\right)}{E_{x}^{l}-E_{x}^{k}}$$

## Appendix C. Mean Energy in a Born–Huang-Like Basis

The mean energy of a 3D system with a Hamiltonian H(x,y,z) is given by,

(A15) $$\langle \hat{E}\rangle =\;\int \int \int \;dxdydz\Psi^{*}\left(x,y,z\right)H\left(x,y,z\right)\Psi \left(x,y,z\right),$$

which in terms of the Hamiltonian in Equation (3) and the wavefunction expansion in Equation (6) can be written as:

(A16) $$\begin{array}{ccc} \langle \hat{E}\rangle & = & \sum_{k,l}^{\infty }\int dx\chi^{*k}\left(x,t\right)\int dy\int dz\phi_{x}^{*k}\left(y,z\right)\left(T_{x}+V\left(x\right)\right)\phi_{x}^{l}\left(y,z\right)\chi^{l}\left(x,t\right)\\ & + & \sum_{k,l}^{\infty }\int dx\chi^{*k}\left(x,t\right)\int dy\int dz\phi_{x}^{*k}\left(y,z\right)H\left(y,z\right)\phi_{x}^{l}\left(y,z\right)\chi^{l}\left(x,t\right). \end{array}$$

Introducing now Equation (5) and Equation (A2) into Equation (A16), we finally get:

(A17) $$\begin{array}{ccc} \langle \hat{E}\rangle & = & \sum_{k,l}^{\infty }\int dx\chi^{*k}\left(x,t\right)\int dy\int dz\phi_{x}^{*k}\left(y,z\right)(T_{x}\chi^{l}\left(x,t\right)\phi_{x}^{l}\left(y,z\right)-\frac{1}{2}\chi^{l}\left(x,t\right)\frac{\partial^{2}}{\partial x^{2}}\phi_{x}^{l}\left(y,z\right)\\ & - & \frac{\partial }{\partial x}\chi^{l}\left(x,t\right)\frac{\partial }{\partial x}\phi_{x}^{l}\left(y,z\right)+V\left(x\right)\chi^{l}\left(x,t\right)\phi_{x}^{l}\left(y,z\right))+\sum_{k}\int dx\chi^{*k}\left(x,t\right)E_{x}^{k}\left(y,z\right)\chi^{k}\left(x,t\right)\\ & = & \sum_{k}^{\infty }\int dx\chi^{*k}\left(x,t\right)T_{x}\chi^{k}\left(x,t\right)+\sum_{k}\int dx\chi^{*k}\left(x,t\right)E_{x}^{k}\left(y,z\right)\chi^{k}\left(x,t\right)+\sum_{k}^{\infty }\int dx\chi^{*k}\left(x,t\right)V\left(x\right)\chi^{k}\left(x,t\right)\\ & + & \sum_{k,l}^{\infty }[-\frac{1}{2}\chi^{*k}\left(x,t\right)\int dy\int dz\phi_{x}^{*k}\left(y,z\right)\frac{\partial^{2}}{\partial x^{2}}\phi_{x}^{l}\left(y,z\right)\\ & - & \chi^{*k}\left(x,t\right)\int dy\int dz\phi_{x}^{*k}\left(y,z\right)\frac{\partial }{\partial x}\phi_{x}^{l}\left(y,z\right)\frac{\partial }{\partial x}]\chi^{l}\left(x,t\right)\\ & = & \sum_{k}^{\infty }\int dx\chi^{*k}\left(x,t\right)[\left(T_{x}+E_{x}^{k}\left(y,z\right)+V\left(x\right)\right)\chi^{k}\left(x,t\right)+\sum_{l}^{\infty }(-\frac{1}{2}\int dy\int dz\phi_{x}^{*k}\left(y,z\right)\frac{\partial^{2}}{\partial x^{2}}\phi_{x}^{l}\left(y,z\right)\\ & - & \int dy\int dz\phi_{x}^{*k}\left(y,z\right)\frac{\partial }{\partial x}\phi_{x}^{l}\left(y,z\right)\frac{\partial }{\partial x})\chi^{l}\left(x,t\right)]\\ & = & \sum_{k=1}^{\infty }\int dx\chi^{*k}\left(x,t\right)\left[\left(T_{x}+E_{x}^{k}+V\left(x\right)\right)\chi^{k}\left(x,t\right)-\sum_{l=1}^{\infty }\left(S^{kl}\left(x\right)+F^{kl}\left(x\right)\frac{\partial }{\partial x}\right)\chi^{l}\left(x,t\right)\right]. \end{array}$$

## Appendix D. Definition of the Nano-Constriction in Figure 1

For our numerical simulations in Section 3, we considered a typical constriction where $L_{1}\left(x\right)$ and $L_{2}\left(x\right)$ in Equation (14) are defined as:

(A18a) $$L_{1}\left(x\right)=A\left[{\left(1+\exp\left(\frac{x-a_{1}}{\gamma }\right)\right)}^{-1}+{\left(1+\exp\left(\frac{-x+a_{2}}{\gamma }\right)\right)}^{-1}\right]+B,$$

(A18b) $$L_{2}\left(x\right)=A\left[{\left(1+\exp\left(\frac{-x+a_{1}}{\gamma }\right)\right)}^{-1}+{\left(1+\exp\left(\frac{x-a_{2}}{\gamma }\right)\right)}^{-1}\right]-A,$$

where γ defines the sharpness of the constriction, A and B define the maximum and the minimum width of the constriction, respectively max[L(x)]=B+A and min[L(x)]=B−A, and $a_{1}$ and $a_{2}$ define the length of the constriction $L_{c}=a_{2}-a_{1}$.
